# Supplementary material for: Blooming plant species diversity patterns in two adjacent Costa Rican highland ecosystems
Source: PeerJ. 2023 Jan 12;11:e14445. doi: 10.7717/peerj.14445 (PMC9840854; doi:10.7717/peerj.14445)
Supplement: Supplemental Information 1 [file peerj-11-14445-s001.docx]

Supplementary material


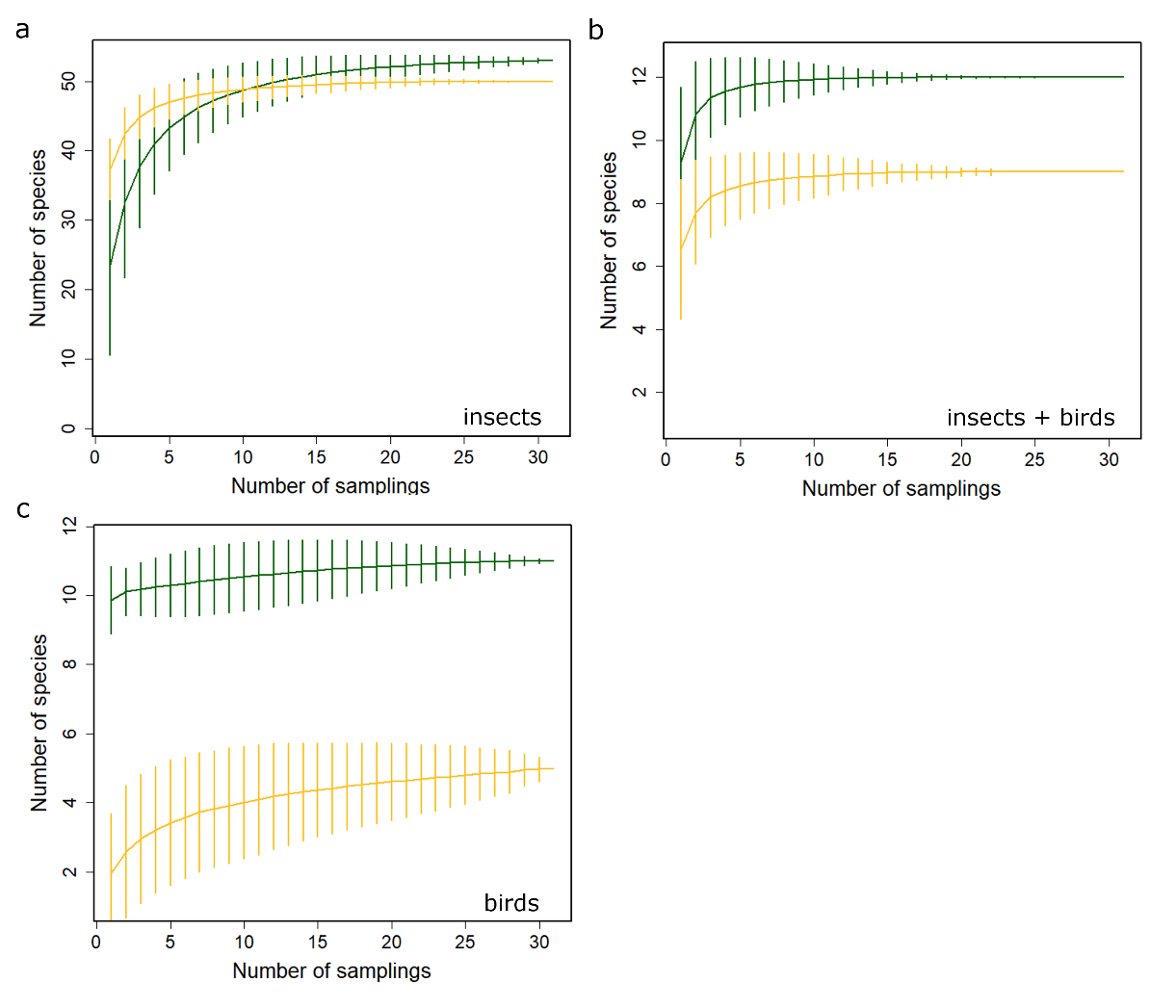


Fig. S1. Sample-based rarefaction curves with 95% confidence intervals for flowering plant species in the montane forest (green lines) and the paramo (golden lines): a- flowering plants visited by insects, b- flowering plants visited by insects+birds, and c- flowering plants visited by birds.


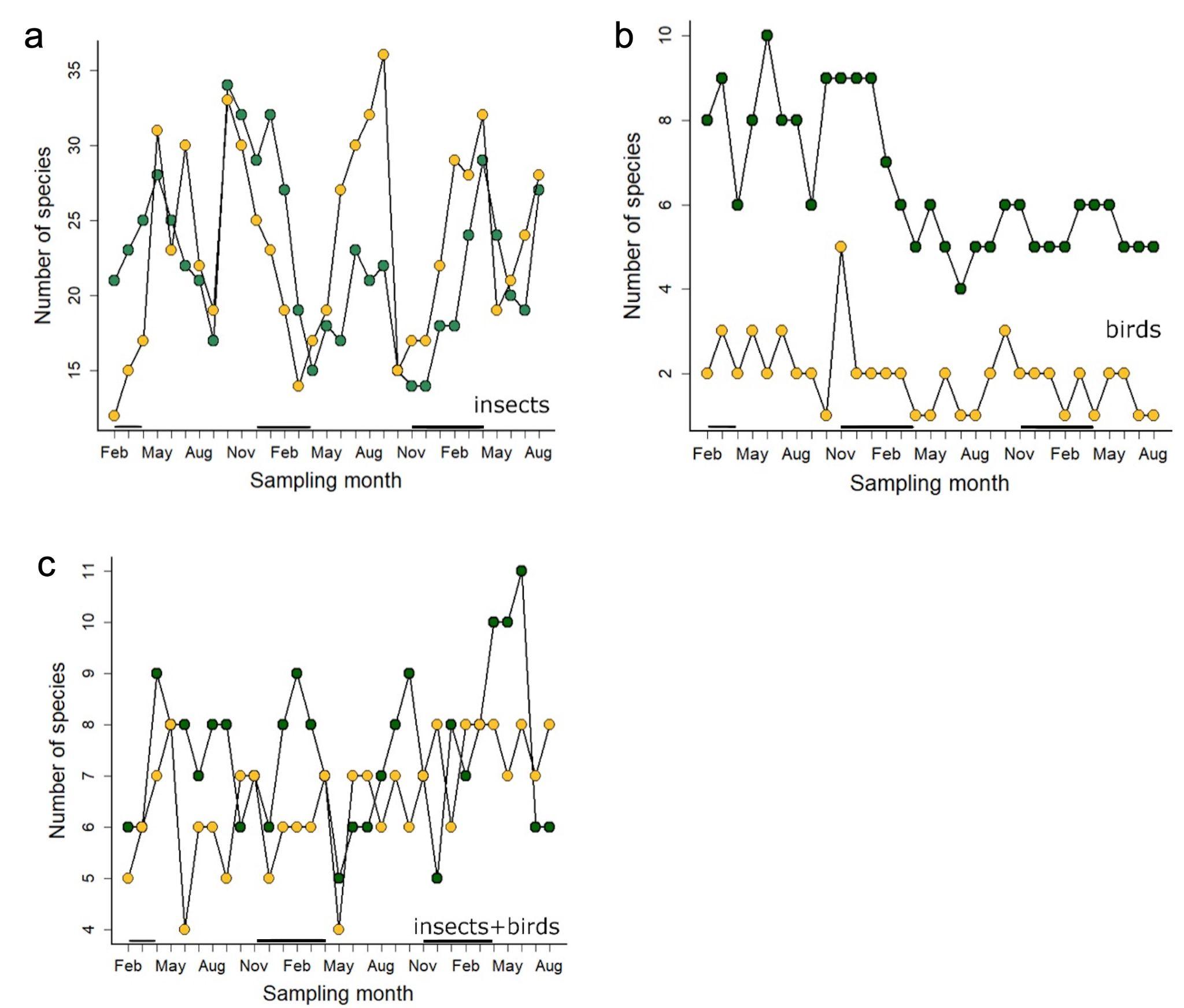


Figure S2. Number of flowering plant species in the Montane Forest (green dots) and the Paramo (goldenrod dots) counted during the study period (2019-2021): a- flowering plants visited by insects, b- flowering plants visited by birds c- flowering plants visited by insects+birds. The solid black lines just above the x axis indicate the dry season months.


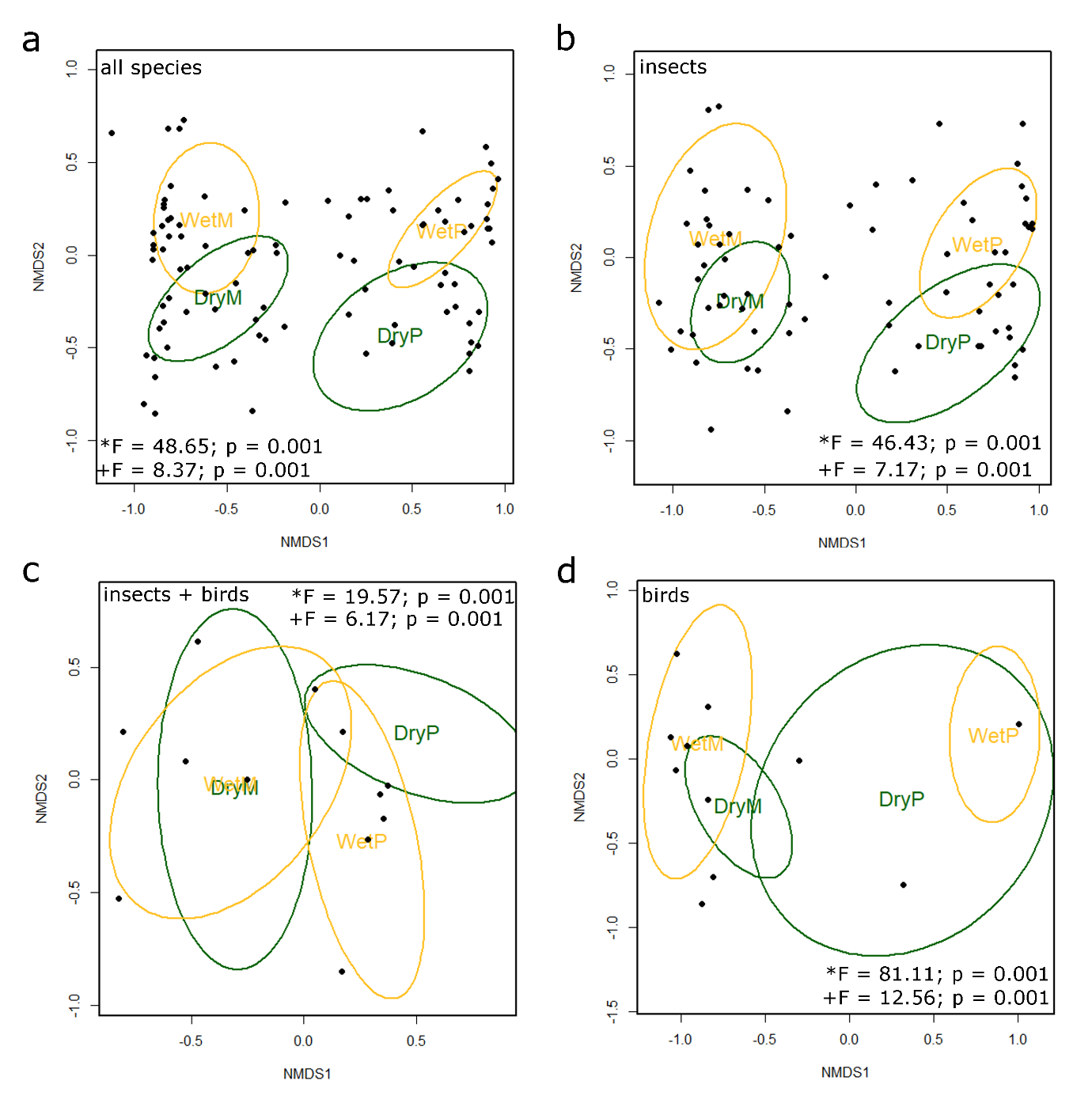


Figure S3. NMDS analyses on flowering species composition between the montane forest and the paramo, and dry and wet seasons: a- all flowering plant species, b- insect pollinated plants, c- insect+bird pollinated plants, d- bird pollinated plants. The * before the F value indicates the results of the ANOVA comparing sites (M- Montane Forest, and P- paramo), and the + corresponds to the comparison between seasons (Dry and Wet).


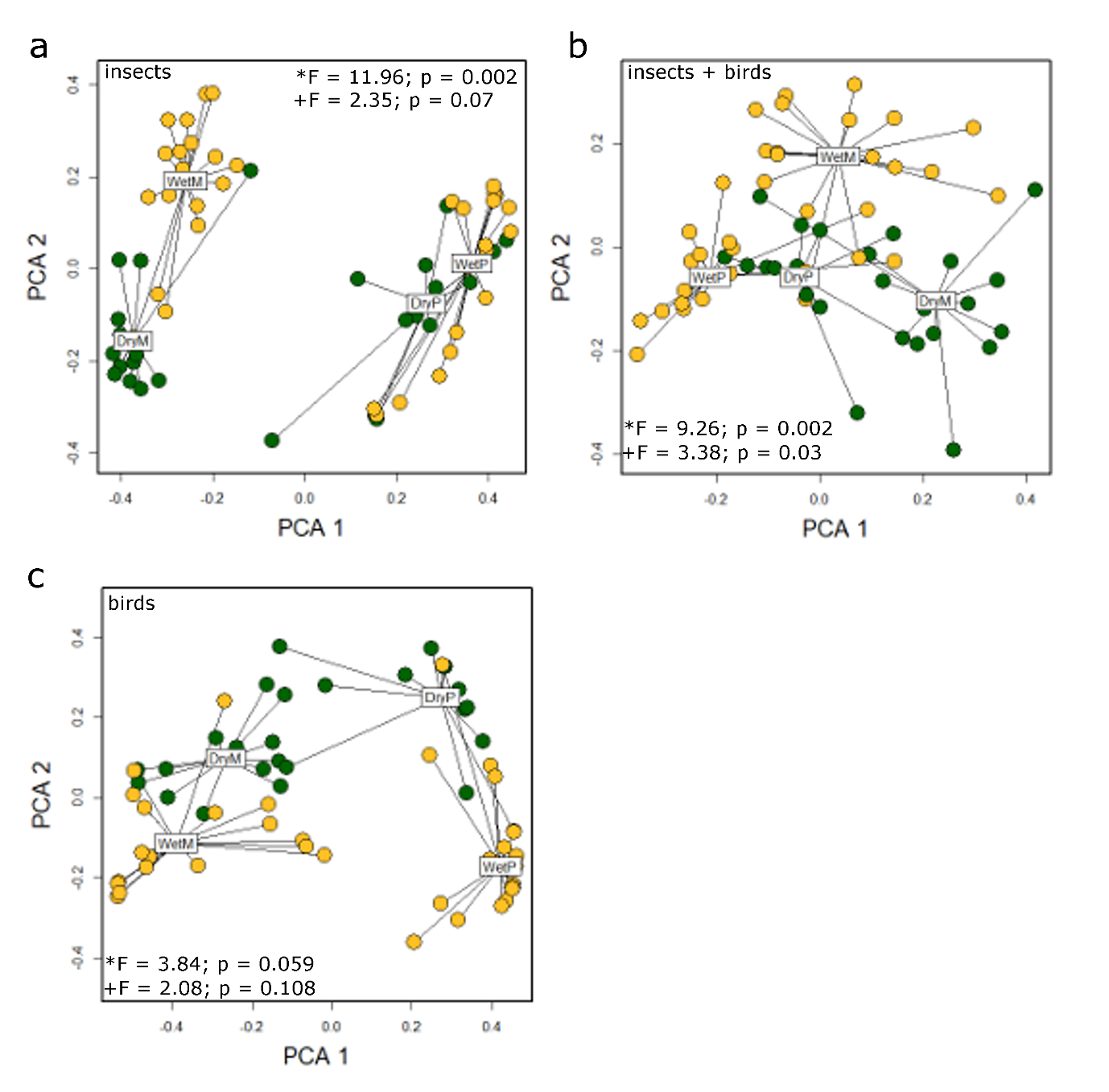


Figure S4. Comparison of *Beta* diversity between sites and seasons. (a) Beta diversity for insect pollinated plants, (b) insect+bird pollinated plants, and (c) bird pollinated plants. The * before the F value indicates the results of the ANOVA comparing sites (M- Montane Forest, and P-Paramo), and the + corresponds to the comparison between seasons (Dry and Wet).

Tables

Table S1. Non-parametric PERMANOVA based on Bray–Curtis distances for insect+bird pollinated, insect-pollinated and bird-pollinated blooming plants at two sites (Montane Forest and the Paramo), two seasons (dry and rainy), and their interaction.

| **Insect+bird pollinated plants** (MSD/Bray – Stress = 0.95) | | | | | |
| --- | --- | --- | --- | --- | --- |
| Factor | df | SS | R2 | F | P |
| Site | 1 | 1.30 | 0.20 | 19.57 | 0.001 |
| Season | 1 | 0.96 | 0.15 | 14.42 | 0.001 |
| Site*season | 1 | 0.41 | 0.06 | 6.17 | 0.001 |
| Residual | 58 | 3.84 | 0.59 |  |  |
| Total | 61 | 6.51 | 1.00 |  |  |
| **Insect-pollinated plants** (MSD/Bray – Stress = 0.98) | | | | | |
| Factor | df | SS | R2 | F | P |
| Site | 1 | 5.81 | 0.37 | 46.43 | 0.001 |
| Season | 1 | 1.65 | 0.11 | 13.21 | 0.001 |
| Site*season | 1 | 0.90 | 0.06 | 7.17 | 0.001 |
| Residual | 58 | 7.26 | 0.46 |  |  |
| Total | 61 | 15.63 | 1.00 |  |  |
| **Bird-pollinated plants** (MSD/Bray – Stress = 0.99) | | | | | |
| Factor | df | SS | R2 | F | P |
| Site | 1 | 6.53 | 0.48 | 81.11 | 0.001 |
| Season | 1 | 1.36 | 0.10 | 16.84 | 0.001 |
| Site*season | 1 | 1.01 | 0.07 | 12.56 | 0.001 |
| Residual | 58 | 4.67 | 0.34 |  |  |
| Total | 61 | 13.58 | 1.00 |  |  |
